# Editorial: The cognitive basis for decision making under risk and uncertainty: research programs & controversies

**DOI:** 10.3389/fpsyg.2025.1546461

**Published:** 2025-02-27

**Authors:** Samuel Shye, Riccardo Viale

**Affiliations:** ^1^Department of Philosophy, Economics & Political Science (PEP), Hebrew University of Jerusalem, Jerusalem, Israel; ^2^Department of Psychology, Hebrew University of Jerusalem, Jerusalem, Israel; ^3^Department of Economics, University of Milano-Bicocca, Milan, Italy

**Keywords:** expected utility theory (EUT), adaptive heuristics, ecological bounded rationality, challenge theory of decision under risk, dual system theory, enactive problem solving

This volume showcases alternative research strategies in decision-making under risk and presents thought-provoking decision problems. The dominant approach in this research domain, rooted in Expected Utility Theory (EUT), emphasizes identifying functions that account for deviations from EUT, typically overlooking the cognitive processes involved. This limits its explanatory power and offers little guidance for improving decision-making. Nonetheless, the insights and terminology introduced by Kahneman and Tversky (1979) and Tversky and Kahneman (1992), who advanced this approach, remain influential, as reflected throughout this Research Topic.

An alternative approach, developed by Simon (1981, 1982)^*^ and expanded by Gigerenzer et al. (1999) and Gigerenzer and Selten (2001), focuses on simple adaptive heuristics and ecological bounded rationality. It aims to improve decision theory by identifying cognitive processes that allow satisfactory choices when perfect optimization is not possible. Moreover, it focuses on the features of the environment, which are often characterized by uncertainty, complexity, and ambiguity. This volume contributes to this perspective by exploring attention allocation in heuristic decisions (Chapter 1), proposing a free parameter for error adjustment in heuristic choices (Chapter 3), examining the role of heuristics in biased choices (Chapter 4), and identifying heuristic elements in intuitive decision-making (Chapter 2).

A novel approach, Challenge Theory (CT; Shye and Haber, 2020a,b), integrates elements from the two approaches mentioned above. CT conceptualizes cognitive decision-making as two sequential thought processes: the heuristic (System 1), which reacts to probabilities and defines the default option, and the deliberate (System 2), which reevaluates the default and may opt for the “bold” alternative. Thus, in loss contexts, CT redefines the risky option as the “default” and the safe option as “bold,” providing a streamlined, one-parameter explanation for the psychological effects—the certainty effect, Allais Paradox, reflection, overweighting of low probabilities, and loss aversion—identified by Kahneman and Tversky as deviations from EUT. Initial experiments suggest that CT outperforms traditional economic models. Key elements of CT are echoed in various chapters of this Research Topic, such as the two-system approach (Chapter 4), the possible prominence of probabilities over outcomes (Chapter 2), and heuristics as a starting point in a sequential cognitive decision process (Chapter 1).

A more radical approach connected to ecological bounded rationality is introduced by Viale (2024) and Viale et al. (2023a), who integrate Simon and Newell's (1971) problem-solving framework into the emerging research paradigm of embodied cognition (Viale et al., 2023b). Simon (1986) emphasizes the centrality of problem-solving, distinguishing it from decision-making, which he considers a subsequent phase. According to Simon, the essence of rationality lies in the ability to adapt, with adaptation relying more on external environmental interactions than on internal cognition. Behavior aligns with external objectives, revealing systemic constraints on adaptation. Simon (1981) highlights the critical role of environmental feedback in shaping actions and narrowing the problem space—the set of potential situations to explore for solutions. In the context of embodied cognition, the problem space represents solutions enabled by environmental affordances (Viale, 2024). This perspective of enactive problem-solving bypasses the analytic phase of decision-making, reducing reliance on symbolic representation and focusing on iterative, action-driven feedback processes.

Below are brief descriptions of each chapter in this volume:

1. To enhance the explanatory power of decision theory, Zilker and Pachur advocate shifting the focus toward how imbalances in attention allocation, rather than distorted risk perceptions, shape decision-making. This approach offers deeper insights into how preferences are formed and holds promise for refining current heuristic models of risky decision-making.

2. Erev and Marx argue that the mainstream assumption of separating judgment from decision-making leads to oversensitivity to rare events. Additionally, the belief that providing a full description of incentives replaces judgment and past experiences overlooks the significant role past experiences play in decision-making. They propose that decision processes are more akin to machine learning classification, where patterns are recognized, rather than the traditional two-stage model of judgment and utility calculation.

3. Spiliopoulos and Hertwig propose that heuristic models, traditionally deterministic and parameter-free, can be enhanced by incorporating an error mechanism to account for stochastic choice. This modification introduces only a single free parameter while preserving the core cognitive processes of the original models. They explore different error mechanisms and examine how this adjustment influences comparisons between heuristics and more complex, parameter-rich models.

4. Korniychuk and Uhlmann model how automatic preferences influence decision-making during problem-solving through trial and error. They show that biases are beneficial early on but detrimental later. Timely “rebiasing”—reversing initial preferences—can lead to superior outcomes. This approach offers a strategic alternative to correcting biases, suggesting that organizations can improve performance by changing key decision-makers rather than eliminating biases entirely.

5. Gigerenzer and Garcia-Retamero find that men's widespread reluctance to take DNA tests to determine biological fatherhood is empirically linked to risk aversion. They conclude that this reluctance stems from anticipated regret: men fear potential embarrassment, if non-paternity is discovered; or potential strain on their relationship, if paternity is confirmed.

6. Loued-Khenissi and Corradi-Dell'Acqua investigated people's choices between two treatment options for serious diseases: a sure but mild improvement (sure option) or a riskier cure with a given probability of success (risky option). Results revealed a general preference for the riskier option, regardless of whether the recipient was oneself, a loved one, or a stranger. However, this preference diminished as the severity of the disease increased.

7. Two common errors in sequential investment decisions are escalation of commitment—persisting with a failing course of action—and prematurely abandoning a successful one. Doerflinger et al., using an incentivized task, identified three key determinants of escalation: personal responsibility, preference for initial investments, and loss framing. Notably, personal responsibility worsened decision quality, as participants were more likely to reinvest when accountable for prior decisions.

8. Huang and Leung examine how risk aversion influences belief updating, showing that stronger risk aversion leads to more conservative actions and reduces the value of new information. With self-relevant information (e.g., IQ), greater risk aversion leads to more belief change, while with self-irrelevant information, it leads to less belief change. Experimental results support this theory, with implications for persuasion, advertising, and political campaigns.

9. Shang and Liu explored how voice attractiveness influences cooperative behavior in economic games, with voices presented for 2,040 ms. Participants were more likely to invest in partners with attractive voices, confirming the “beauty premium” effect. They also invested more in male partners. Event-related potential (ERP) analysis showed that attractive voices reduced negative feelings after losses, suggesting that voice attractiveness weakens frustration and enhances cooperative behavior during feedback evaluations.

10. Stegmüller et al. examine how natural frequencies, known to aid Bayesian reasoning, perform in scenarios involving joint probabilities of binary events. Using a 2 × 5 × 2 design, they explored different information formats and visualization types. Surprisingly, natural frequencies did not show the same advantage for joint probabilities as in typical Bayesian tasks. The format effect interacted with visualization types, with natural frequencies aiding understanding in some cases (like a double tree) but not in others (like a 2 × 2 table).

## Conclusion

In conclusion, while cognitive-psychological approaches provide a more appropriate framework for understanding human decision-making under risk than Expected Utility Theory (EUT) or its derivatives, Aumann's (2019) thesis remains relevant: people generally make decisions that align with EUT. Indeed, people follow behavioral rules of thumb, which have evolved because they generally promote human goals, that is, accord with EUT. Deviations from EUT typically occur in rare or contrived scenarios that are not subject to evolutionary pressures. As Tversky wryly observed while developing Prospect Theory: “Despite deviations from EUT, humans have managed rather well” (personal communication, Tversky, 1975). Thus, while cognitive-psychological processes undeniably shape decision-making under risk, their outcomes align sufficiently with EUT to ensure human survival.

## Footnotes

^1^When we began planning this project, Shabnam Mousavi was one of our co-editors. Her intelligence, creativity, intellectual independence, and profound knowledge would have been invaluable to the success of this endeavor. Tragically, Shabnam passed away before we could benefit from her contributions. We have strived to honor her vision in shaping this issue, which is dedicated to her memory.
